# Geographic style maps for two-dimensional lattices

**DOI:** 10.1107/S2053273322010075

**Published:** 2023-01-01

**Authors:** Matthew Bright, Andrew I. Cooper, Vitaliy Kurlin

**Affiliations:** a Materials Innovation Factory, University of Liverpool, UK; Universidad del País Vasco, Spain

**Keywords:** two-dimensional lattices, reduced basis, obtuse superbase, isometry, complete invariants, metric tensor, continuity

## Abstract

This paper develops geographic style maps containing two-dimensional lattices in all known periodic crystals parameterized by recent complete invariants. Motivated by rigid crystal structures, lattices are considered up to rigid motion and uniform scaling. The resulting space of two-dimensional lattices is a square with identified edges or a punctured sphere. The new continuous maps show all Bravais classes as low-dimensional subspaces, visualize hundreds of thousands of lattices of real crystal structures from the Cambridge Structural Database, and motivate the development of continuous and invariant-based crystallography.

## Abstract 2

Continuous invariant-based maps visualize for the first time all two-dimensional lattices extracted from hundreds of thousands of known crystal structures in the Cambridge Structural Database.

## 1. Practical motivations for solving the problem of how to continuously classify lattices

This paper for mathematical crystallographers presents applications of the work of Kurlin (2022*b* ▸) written for mathematicians and computer scientists, with proofs of the invariance of map coordinates up to basis choice, and their continuity under perturbations of a basis. A lattice can be considered as a periodic crystal whose atomic motif consists of a single point. In Euclidean space 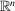 , a lattice 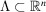 consists of all integer linear combinations of basis vectors *v*_1_, …, *v*_*n*_, which span a primitive unit cell *U* of Λ.

Crystallography traditionally splits crystals into only finitely many classes, for instance by their space-group types. These discrete symmetry-based classifications were suitable for distinguishing highly symmetric crystals manually or simply by eye. Nowadays crystals are simulated and synthesized on an industrial scale. The Cambridge Structural Database (CSD) contains nearly 1.2 million existing crystal structures (Groom *et al.*, 2016 ▸). Crystal structure prediction (CSP) tools generate millions of crystal structures even for a fixed chemical composition (Pulido *et al.*, 2017 ▸), mostly with *P*1 symmetry. Data sets of this size require finer classifications than by 230 crystallographic groups.

**Figure 1:**
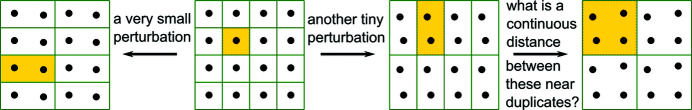
For almost any perturbation of atoms, the symmetry group and any reduced cell (even its volume) discontinuously change, which justifies a continuous classification.

**Figure 2:**
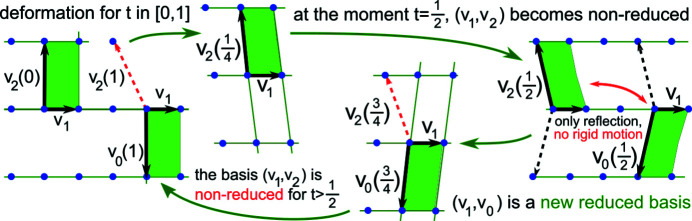
The deformation of the basis *v*_1_ = (1, 0), *v*_2_ = (−*t*, 2) for *t* ∈ [0, 1] defines a continuous loop of lattices. The basis *v*_1_, *v*_2_ is reduced for 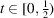 but after 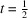 switches to a non-equivalent (up to rigid motion) reduced basis *v*_1_, *v*_0_ = (*t* − 1, −2).

A more important reason for a continuous approach to classifying periodic structures is the inevitability of noise in data. Slight changes in initial simulated or actual crystallization conditions mean that the same crystal can have slightly different X-ray patterns, leading to close but distinct structures. Fig. 1 ▸ shows that a reduced cell cannot be used to continuously quantify a distance between general periodic sets. If we consider only lattices, a similar discontinuity of a reduced basis arises in Fig. 2 ▸.

Consider the family of lattices with the basis *v*_1_ = (1, 0), *v*_2_(*t*) = (−*t*, 2) in Fig. 2 ▸, where the parameter *t* varies continuously in [0, 1]. Since the initial basis *v*_1_ = (1, 0), *v*_2_(0) = (0, 2) and final basis *v*_1_ = (1, 0), *v*_2_(1) = (−1, 2) define identical lattices, this continuous family of lattices is a closed loop in the space of all lattices. For 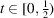 , the given basis *v*_1_ = (1, 0), *v*_2_(*t*) = (−*t*, 2) is reduced by Definition 2.1[Statement definition2.1]. At the critical moment 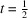 , the lattice has several primitive bases that can be chosen as reduced.

When *t* passes through 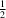 , if we keep the angle between basis vectors continuous, the reduced basis *v*_1_ = (1, 0), 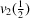 = 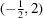 switches to *v*_1_ = (1, 0), 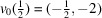 . For any choice at 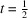 , the basis *v*_1_ = (1, 0), *v*_0_(*t*) = (*t* − 1, − 2) will be a new reduced basis for 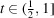 . The above change at 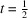 creates the discontinuity because the given bases 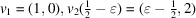 and 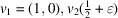 = 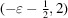 at 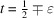 differ only by a small perturbation 2ɛ > 0 in all coordinates but the lattices have the reduced bases *v*_1_, 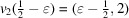 and *v*_1_, 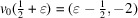 , whose last coordinates differ by 4. These reduced bases cannot be made close by rigid motion because they have opposite anticlockwise angles from *v*_1_ to the longer vector.

One way to call lattices identical (or equivalent) is to ignore deviations of lattice parameters up to a certain threshold. An equivalence gives rise to a justified classification only if this *equivalence relation* (denoted by ∼) satisfies the axioms: (i) reflexivity: any lattice Λ is equivalent to itself, so Λ ∼ Λ; (ii) symmetry: if Λ ∼ Λ′, then Λ′ ∼ Λ; (iii) transitivity: if Λ ∼ Λ′ and Λ′ ∼ Λ′′, then Λ ∼ Λ′′.

**Figure 3:**
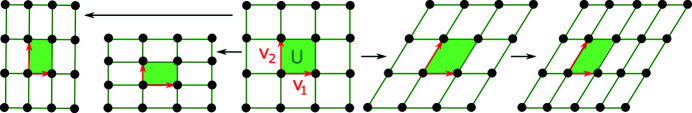
All lattices continuously deform into each other if we allow any small changes.

The transitivity axiom is needed to split lattices into disjoint *equivalence classes*: the class [Λ] consists of all lattices equivalent to Λ, since if Λ is equivalent to Λ′, which is equivalent to Λ′′, all three lattices are in the same class. Past equivalences in the work of Lima-de-Faria *et al.* (1990 ▸) use numerical thresholds to determine a lattice class but, as Fig. 3 ▸ illustrates, all lattices can be made equivalent through sufficiently many slight perturbations up to any positive threshold due to the transitivity axiom.

An alternative mathematical approach classifies lattices by space groups and finer algebraic structures (Nespolo, 2008 ▸). Since crystal structures are determined as rigid forms, the most practically important equivalence of crystal structures and their lattices is a *rigid motion*, which in 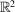 is any composition of translations and rotations. This is the strongest possible equivalence on crystals that are indistinguishable as rigid bodies.

Slightly weaker is equivalence based on *isometry* or congruence, denoted by Λ ≅ Λ′, which is any rigid motion composed of mirror reflections. Even if we fix an equivalence such as isometry, Sacchi *et al.* (2020 ▸) highlight that the key question ‘same or different’ remains unanswered. What is needed is the notion of an *invariant*.

**Figure 4:**
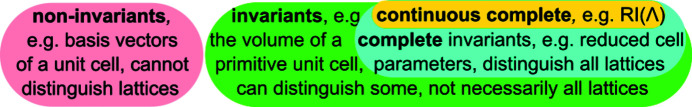
The root invariant RI(Λ) from Definition 3.1[Statement definition3.1] used for mapping crystal structures from the CSD in this paper is a continuous and complete isometry invariant of all two-dimensional lattices.

Definition 1.1 (invariants versus complete invariants) A descriptor *I*, such as a numerical vector, is called an *isometry invariant* of a lattice 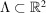 if *I* takes the same value on all isometric lattices: if Λ ≅ Λ′ are isometric then *I*(Λ) = *I*(Λ′), so *I* has no *false negatives*. An isometry invariant *I* is called *complete* (or *injective*) if the converse also holds: if *I*(Λ) = *I*(Λ′) then Λ ≅ Λ′, so *I* distinguishes all non-isometric lattices. Hence a complete invariant *I* has neither false negatives nor false positives (see Fig. 4 ▸).

In a fixed coordinate system, the basis vectors are not isometry invariants as they change under rotation, but the primitive cell area is preserved by isometry. If an invariant *I* takes different values on lattices Λ, Λ′, these lattices are certainly not isometric, while non-invariants cannot help distinguish equivalent objects. For example, isometric lattices Λ ≅ Λ′ can have infinitely many primitive bases. Most isometry invariants allow *false positives* that are non-isometric lattices 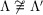 with *I*(Λ) = *I*(Λ′). For instance, infinitely many non-isometric lattices have the same primitive cell area.

Complete invariants are the main goal of all classifications. *Continuous* invariants, which change only slightly under small perturbations of the underlying object, are even better. The dependence of pseudosymmetry on thresholds discussed by Zwart *et al.* (2008 ▸) can be resolved in a continuous way by finding, for any given lattice, its closest higher-symmetry neighbour through continuous invariants as in Problem 1.2[Statement enun1.2].

Problem 1.2 Find a complete isometry invariant *I*(Λ) of any lattice 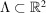 with a metric *d* satisfying all necessary axioms and the new continuity condition below:

(i) First axiom: *d*(Λ, Λ′) = 0 if and only if Λ ≅ Λ′ are isometric;

(ii) Symmetry axiom: *d*(Λ, Λ′) = *d*(Λ′, Λ) for any lattices 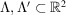 ;

(iii) Triangle axiom: *d*(Λ, Λ′) + *d*(Λ′, Λ′′) ≥ *d*(Λ, Λ′′) for any lattices 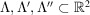 ;

(iv) Lipschitz continuity: there is a constant *C* such that, for any lattices 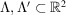 , if corresponding coordinates of their basis vectors differ by at most ɛ > 0, then *d*(Λ, Λ′) ≤ *C*ɛ.

This paper applies a solution of Problem 1.2 from Kurlin (2022*b* ▸) to visualize crystal structures in the CSD on continuous maps. Sections 2 and 3 review the related past work. Section 4 maps hundreds of thousands of crystal structures in the CSD. Section 5 explains the geographical metaphor by mapping the invariant values to a sphere, where every two-dimensional lattice (up to rigid motion and uniform scaling) has unique latitude and longitude coordinates.

## 2. Overview of key concepts and past work on classifications of lattices

Crystallography traditionally uses a conventional cell to uniquely represent any periodic crystal (see Hahn *et al.*, 2016 ▸). In the simpler case of three-dimensional lattices, the cell used is Niggli’s reduced cell (Niggli, 1928 ▸). Since the current paper studies lattices in 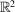 , we give the two-dimensional version obtained from the three-dimensional definition, which is derived as a limit of the reduction conditions for a three-dimensional reduced basis with an orthogonal third vector *v*_3_ whose length becomes infinite. For vectors *v*_1_ = (*a*_1_, *a*_2_) and *v*_2_ = (*b*_1_, *b*_2_) in 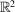 , the determinant of the matrix

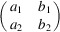

with the columns *v*_1_, *v*_2_ is defined as 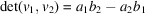 .

Definition 2.1 (reduced cell) For a lattice up to isometry, a basis and its cell *U*(*v*_1_, *v*_2_) are called *reduced* (non-acute) if |*v*_1_| ≤ |*v*_2_| and 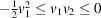 . Up to rigid motion, the conditions are weaker: |*v*_1_| ≤ |*v*_2_| and 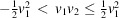 , 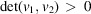 , and the new *special condition* for rigid motion is: if |*v*_1_| = |*v*_2_| then *v*_1_ *v*_2_ ≥ 0.

The new conditions for rigid motion did not appear in the work of de Wolff (2016 ▸) because reduced bases were considered up to isometry including reflections. Any rectangular lattice has a unique (up to rigid motion) reduced cell *a* × *b*, but two ‘potentially reduced’ bases *v*_1_ = (*a*, 0) and *v*_2_ = (0, ± *b*), which are not related by rigid motion for 0 < *a* < *b*. Definition 2.1[Statement definition2.1] chooses only one of these bases, namely *v*_1_ = (*a*, 0) and *v*_2_ = (0, *b*). So 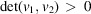 defines a right-handed basis in 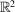 .

Since reduced bases are easy to compute (Křivý & Gruber, 1976 ▸), they can be used to define the discrete metric *d*(Λ, Λ′) taking the same non-zero value (say, 1) for any non-isometric lattices 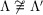 . Discontinuity of a reduced basis up to perturbations was practically demonstrated in the seminal work of Andrews *et al.* (1980 ▸). The introduction of Edelsbrunner *et al.* (2021 ▸) said that ‘There is no method for choosing a unique basis for a lattice in a continuous manner. Indeed, continuity contradicts uniqueness as we can continuously deform a basis to a different basis of the same lattice’; see Fig. 2 ▸ and a formal proof in Widdowson *et al.* (2022 ▸, theorem 15). Since a reduced basis is discontinuous under perturbations, then so is any metric on these reduced bases.

Important advances were made (Andrews & Bernstein, 1988 ▸, 2014 ▸; McGill *et al.*, 2014 ▸; Andrews *et al.*, 2019*a* ▸; Bernstein *et al.*, 2022 ▸) by analysing complicated boundary cases where cell reductions can be discontinuous. Since these advances are specialized for 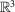 , we refer the reader to another paper (Bright *et al.*, 2021 ▸) for a detailed review of reduced bases for three-dimensional lattices.

**Figure 5:**
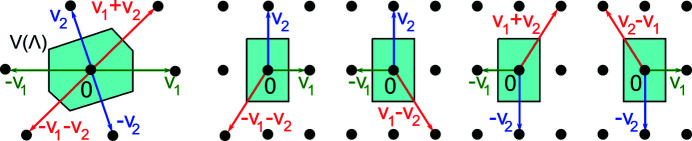
Left: a generic two-dimensional lattice has a hexagonal Voronoi domain with an obtuse superbase *v*_1_, *v*_2_, *v*_0_ = −*v*_1_ − *v*_2_, which is unique up to permutations and central symmetry. Other pictures: isometric superbases for a rectangular Voronoi domain.

Another way to represent a lattice 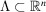 is by its Wigner–Seitz cell (Wigner & Seitz, 1933 ▸) or Voronoi domain *V*(Λ) consisting of all points 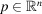 that are closer to the origin 0 ∈ Λ than to all other points of Λ (Fig. 5 ▸). Though *V*(Λ) uniquely determines Λ up to rotations, almost any tiny perturbation of a rectangular lattice Λ converts the rectangular domain *V*(Λ) into a hexagon. Hence all combinatorial invariants (numbers of vertices or edges) of *V*(Λ) are discontinuous, similarly in higher dimensions.

However, comparing Voronoi domains as geometric shapes by optimal rotation (Mosca & Kurlin, 2020 ▸) around a common centre led to two continuous metrics on lattices up to rigid motion and uniform scaling. The minimization over infinitely many rotations was resolved only by finite sampling, so the exact computation of these metrics is still open. Similar computational difficulties remain for stronger isometry invariants of general periodic sets (Anosova & Kurlin, 2021*a* ▸,*b* ▸, 2022*a* ▸,*b* ▸; Smith & Kurlin, 2022 ▸).

Another attempt to produce computable metrics was to consider distance-based invariants (Widdowson *et al.*, 2022 ▸; Widdowson & Kurlin, 2022 ▸) whose completeness was proved for generic crystals. These invariants helped establish the crystal isometry principle by experimentally checking that all periodic crystal structures from the CSD remain non-isometric after forgetting all chemical information. This principle implies that all periodic crystals can be studied in the common crystal isometry space (CRISP) whose version for two-dimensional lattices is the lattice isometry space 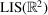 .

Though the paper by Conway & Sloane (1992 ▸) 30 years ago aimed for continuous invariants of three-dimensional lattices, no formal proofs were given even for the isometry invariance. This past work for three-dimensional lattices has been corrected and extended by Kurlin (2022*a* ▸).

Kurlin (2022*b* ▸, proposition 3.10) proves that a reduced basis from Definition 2.1[Statement definition2.1] is unique (also in the case of rigid motion) and all reduced bases are in a 1–1 correspondence with obtuse superbases, which are easier to visualize, especially for *n* ≤ 3.

Definition 2.2 (superbase, conorms pij) For any basis *v*_1_, …, *v*_*n*_ in 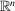 , the *superbase* *v*_0_, *v*_1_, …, *v*_*n*_ from Conway & Sloane (1992 ▸) includes the vector 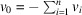 . The *conorms* *p*_*ij*_ = −*v*_*i*_ *v*_*j*_ are the negative scalar products of the vectors. The superbase is called *obtuse* if all *p*_*ij*_ ≥ 0, so all angles between the vectors *v*_*i*_, *v*_*j*_ are non-acute for distinct indices *i*, *j* ∈ {0, 1, …, *n*}. The obtuse superbase is *strict* if all *p*_*ij*_ > 0.

Definition 2.2[Statement definition2.2] uses the conorms *p*_*ij*_ from Conway & Sloane (1992 ▸), which were also known as negative Selling parameters (Selling, 1874 ▸) and Delaunay parameters (Delaunay *et al.*, 1934 ▸). Lagrange (1773 ▸) proved that the isometry class of any lattice 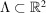 with a basis *v*_1_, *v*_2_ is determined by the *positive quadratic form*


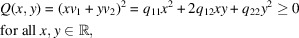

where 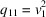 , *q*_12_ = *v*_1_ *v*_2_, 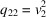 . The triple 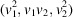 is also called a metric tensor of (a basis of) Λ. Any *Q*(*x*, *y*) has a reduced (non-acute) form with 0 < *q*_11_ ≤ *q*_22_ and −*q*_11_ ≤ 2*q*_12_ ≤ 0, which is equivalent to reducing a basis up to isometry.

The bases *v*_1_ = (3, 0), 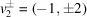 generate the mirror images not related by rigid motion, but define the same form *Q* = 9*x* ^2^ − 6*xy* + 5*y* ^2^ satisfying the reduction conditions above. So quadratic forms do not distinguish mirror images (enantiomorphs). Hence the new conditions for the rigid motion were needed in Definition 2.1[Statement definition2.1].

Motivated by the non-homogeneity of the metric tensor (two squared lengths and scalar product), Delaunay (1937 ▸) proposed the homogeneous parameters 

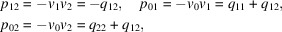

called conorms by Conway & Sloane (1992 ▸) (see Definition 2.2[Statement definition2.2]). Then any permutation of superbase vectors satisfying *v*_0_ + *v*_1_ + *v*_2_ = 0 changes *p*_12_, *p*_01_, *p*_02_ by the same permutation of indices. For example, swapping *v*_1_, *v*_2_ is equivalent to swapping *p*_01_, *p*_02_.

Delaunay’s reduction (Delaunay *et al.*, 1973 ▸) proved the key existence result: any lattice in dimensions 2 and 3 has an obtuse superbase with all *p*_*ij*_ ≥ 0. Section 3 further develops the Delaunay parameters to show in Section 4 how millions of lattices from real crystal structures in the CSD are distributed in continuous spaces of lattices.

## 3. Homogeneous complete invariants of two-dimensional lattices up to four equivalences

This section provides a reminder of the lattice classifications in Theorem 3.4[Statement theorem3.4] based on the recent invariants introduced in Definitions 3.1[Statement definition3.1] and 3.2[Statement definition3.2] from Kurlin (2022*b* ▸, sections 3–4).

Definition 3.1 [sign(Λ) and root invariants RI, RIo] Let *B* = {*v*_0_, *v*_1_, *v*_2_} be any obtuse superbase of a lattice 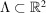 . If Λ is mirror-symmetric (achiral), set sign(Λ) = 0. Otherwise *v*_0_, *v*_1_, *v*_2_ have different lengths and no right angles, and hence can be ordered so that |*v*_1_| < |*v*_2_| < |*v*_0_|. Let sign(Λ) be the sign of 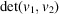 of the matrix with the columns *v*_1_, *v*_2_. The *root invariant* RI(Λ) is the triple of the root products 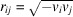 , which have original units of vector coordinates such as ångströms and are ordered by their size for distinct indices *i*, *j* ∈ {0, 1, 2}. The *oriented root invariant* RI^o^(Λ) is RI(Λ) with sign(Λ) as a superscript, which we skip if sign(Λ) = 0.

We assume that *r*_*ij*_ = *r*_*ji*_. If |*v*_1_| < |*v*_2_| < |*v*_0_|, then *r*_12_ < *r*_01_ < *r*_02_. If some *v*_*i*_, *v*_*j*_ have equal lengths, then *r*_*ik*_ = *r*_*jk*_ for *k* ≠ *i*, *j*. Writing RI(Λ) = (*r*_12_, *r*_01_, *r*_02_) means that |*v*_1_| ≤ |*v*_2_| ≤ |*v*_0_| for a suitable indexing of obtuse superbase vectors *v*_0_, *v*_1_, *v*_2_.

Kurlin (2022*b* ▸, lemma 3.8) proved that RI(Λ) is an isometry invariant of Λ, independent of an obtuse superbase *B* because an obtuse superbase of Λ is unique up to isometry, also up to rigid motion for non-rectangular lattices. This uniqueness was missed by Conway & Sloane (1992 ▸) and actually fails in 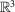 (see Kurlin, 2022*a* ▸).

**Figure 6:**
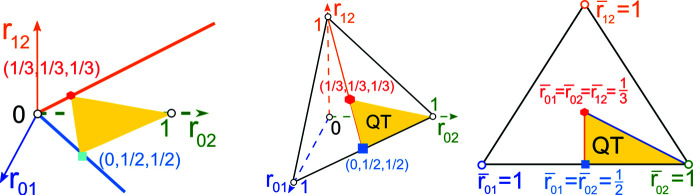
Left: the triangular cone TC = { 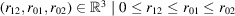 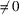 } is the space of all root invariants, see Definition 3.1[Statement definition3.1]. Middle: TC projects to the quotient triangle QT representing all two-dimensional lattices up to isometry and uniform scaling. Right: QT is parameterized by 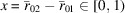 and 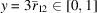 .

**Figure 7:**
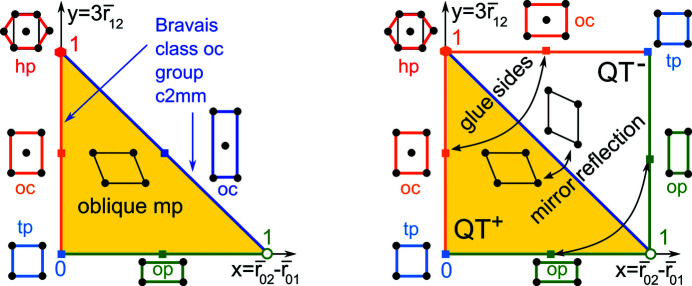
Left: all projected invariants PI(Λ) live in the quotient triangle QT parameterized by 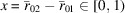 and 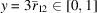 . Right: mirror images (enantiomorphs) of any oblique lattice are represented by a pair (*x*, *y*) ↔ (1 − *y*, 1 − *x*) in the quotient square QS = QT^+^ ∪ QT^−^ symmetric in the diagonal *x* + *y* = 1.

Definition 3.2 (projected invariants PI, PIo) The root invariants of all lattices 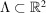 live in the triangular cone TC in Fig. 6 ▸. The triangular projection TP: TC → QT divides each coordinate by the *size* σ(Λ) = *r*_12_ + *r*_01_ + *r*_02_ and projects RI(Λ) to 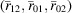 in the quotient triangle QT in Fig. 7 ▸. This triangle can be visualized as the isosceles right-angled triangle 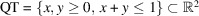 parameterized by 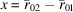 and 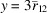 . The resulting pair PI(Λ) = (*x*, *y*) is the *projected invariant*. The oriented invariant PI^o^(Λ) is obtained by adding the superscript sign(Λ).

All oriented projected invariants PI^o^(Λ) with sign(Λ) live in a union of two quotient triangles QT^+^ ∪ QT^−^. These triangles should be glued along the common subspace of mirror-symmetric lattices (all non-oblique lattices 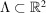 ), whose PI(Λ) belong to the boundary of QT. Fig. 7 ▸ (right) glues the hypotenuses of QT^±^ and indicates how to glue the remaining sides. We get a punctured sphere due to the excluded vertex (1, 0).

**Figure 8:**
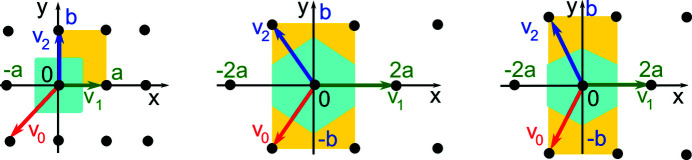
Left: any rectangular lattice Λ with a unit cell *a* × *b* has the obtuse superbase *B* with *v*_1_ = (*a*, 0), *v*_2_ = (0, *b*), *v*_0_ = (−*a*, −*b*), see Example 3.3 (op). Other lattices Λ have a rectangular cell 2*a* × 2*b* and an obtuse superbase *B* with *v*_1_ = (2*a*, 0), *v*_2_ = (−*a*, *b*), *v*_0_ = (−*a*, − *b*). Middle: RI(Λ) = 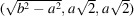 , *a* ≤ *b* ≤ 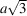 . Right: RI(Λ) = 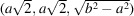 , 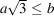 , see Example 3.3 (oc).

*Example 3.3 (subspaces of Bravais classes in QT).*

(tp) The square lattice 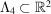 with a unit cell *a* × *a* has RI(Λ_4_) = (0, *a*, *a*) ∈ TC projected by TP to 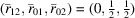 . By Definition 3.2[Statement definition3.2] the projected invariant 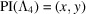 = 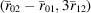 = 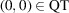 [see Fig. 7 ▸ (left)]. So the Bravais class (tp) of all square (tetragonal) lattices 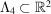 is represented by the bottom-left vertex (0, 0) in the quotient triangle QT, identified with the top-right vertex of the quotient square QS in Fig. 7 ▸ (right).

(hp) The hexagonal lattice Λ_6_ with a minimum inter-point distance *a* has the root invariant 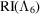 = 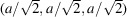 projected by TP to 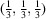 . The projected invariant is PI(Λ_6_) = (*x*, *y*) = (0, 1) ∈ QT [see Fig. 7 ▸ (left)]. The Bravais class (hp) of all hexagonal lattices 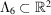 is represented by the top-left vertex (0, 1) in the quotient triangle QT, identified with the bottom-right vertex of the quotient square QS.

(op) Any rectangular lattice Λ with a unit cell *a* × *b* for 0 < *a* < *b* has the obtuse superbase *v*_1_ = (*a*, 0), *v*_2_ = (0, *b*), *v*_0_ = (−*a*, −*b*) [see Fig. 8 ▸ (left)]. Then RI(Λ) = (0, *a*, *b*) and 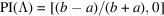 belongs to the horizontal side of QT, which represents the Bravais class (op). We approach the excluded vertex (1, 0) as *b* → +∞.

(oc) Any centred rectangular lattice Λ with a conventional unit cell 2*a* × 2*b* for 0 < *a* < *b* has the obtuse superbase *v*_1_ = (2*a*, 0), *v*_2_ = (−*a*, *b*), *v*_0_ = (−*a*, −*b*) (see Fig. 8 ▸). Then r_01_ = 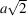 = *r*_02_ and *r*_12_ = 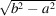 . If 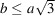 , then RI(Λ) = 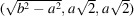 and 

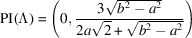

belongs to the vertical (orange) edge of QT. This vertical edge is the shortest straight-line path between the vertices (*x*, *y*) = (0, 0) representing the tetragonal and hexagonal Bravais classes, where *a* = *b* and 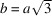 , respectively. Hence the subspace of centred rectangular lattices for 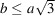 can be considered as having the symmetries of both hexagonal and square lattices. If 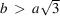 , then RI(Λ) = 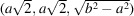 and 

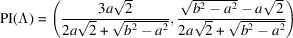

belongs to the hypotenuse *x* + *y* = 1 of the triangle QT. The open vertical edge and open hypotenuse of QT represent the Bravais class oc of all centred rectangular lattices.

The companion paper (Kurlin, 2022*b* ▸) proves the following classifications of two-dimensional lattices up to four equivalences, fulfilling the invariance and completeness conditions.

Theorem 3.4 [proved by Kurlin (2022b, theorem 4.2, corollary 4.6)] For a lattice 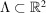 ,

(*a*) the invariant RI(Λ) uniquely identifies Λ up to isometry,

(*b*) the invariant RI^o^(Λ) uniquely identifies Λ up to rigid motion,

(*c*) the invariant PI(Λ) uniquely identifies Λ up to isometry and uniform scaling,

(*d*) the invariant PI^o^(Λ) uniquely identifies Λ up to rigid motion and uniform scaling.

Each part in Theorem 3.4[Statement theorem3.4] can be rephrased as a two-directional criterion. For example, part (*a*): any lattices 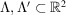 are isometric if and only if RI(Λ) = RI(Λ′). The first (*only if*) direction means that if Λ ≅ Λ′ are isometric, then RI(Λ) = RI(Λ′), so RI(Λ) is an isometry invariant taking the same value on all isometric lattices. The second (*if*) direction means that if RI(Λ) = RI(Λ′), then Λ ≅ Λ′ are isometric.

## 4. Mapping millions of two-dimensional lattices extracted from crystal structures in the CSD

For any periodic crystal structure from the CSD, which has full geometric data of its lattice 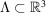 , we extract three two-dimensional lattices generated by three pairs {*v*_2_, *v*_3_}, {*v*_1_, *v*_3_}, {*v*_1_, *v*_2_} of given basis vectors of Λ. So the CSD provides a huge collection of 2.6 million two-dimensional lattices, which our reduction approach maps to the triangle QT in under 1 h on a standard laptop.

**Figure 9:**
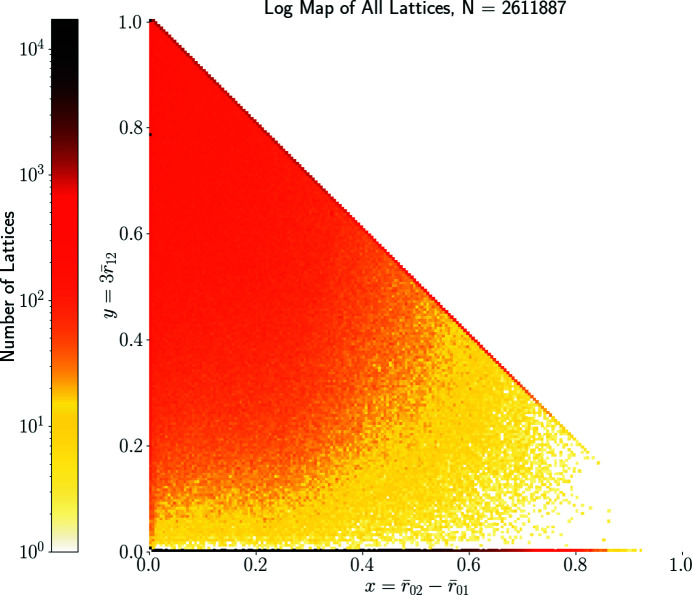
The heat map in QT of all two-dimensional lattices extracted from 870 000+ crystal structures in the CSD. The colour of each pixel indicates (on the logarithmic scale) the number of lattices whose projected invariant 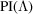 = 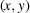 = 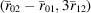 belongs to this pixel. The darkest pixels represent rectangular lattices on the bottom edge of QT.

Fig. 9 ▸ shows all resulting 2.6 million lattices in QT. Only about 55% of all lattices have Bravais classes oc, op, hp, tp. The remaining 45% of lattices are oblique, with Bravais class mp. These occupy almost the full quotient triangle QT, although we see a somewhat greater density close to subspaces representing higher-symmetry lattices – especially around hexagonal and rectangular centred lattices.

**Figure 10:**
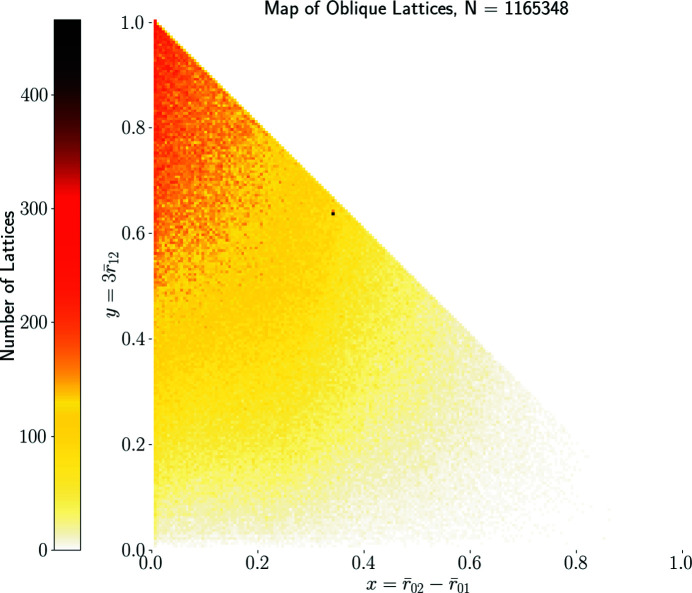
The normal-scale heat map in QT of all two-dimensional oblique lattices from CSD crystals. After removing mirror-symmetric lattices on the boundary of QT, we can better see the tendency towards hexagonal lattices at the top-left corner (0, 1) ∈ QT.

The gap of about two pixels near the horizontal edge in Fig. 9 ▸ corresponds to 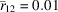 . The relevant lattices have basis vectors *v*_1_, *v*_2_ whose angle is perturbed from 90° by less than 0.03°. The CSD has only 399 such lattices and 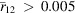 for all but one of them. After removing all non-oblique lattices represented by root invariants along the boundary of QT, the map in Fig. 10 ▸ shows more clearly that all oblique lattices extracted from the CSD occupy the triangle QT without any gaps.

**Figure 11:**
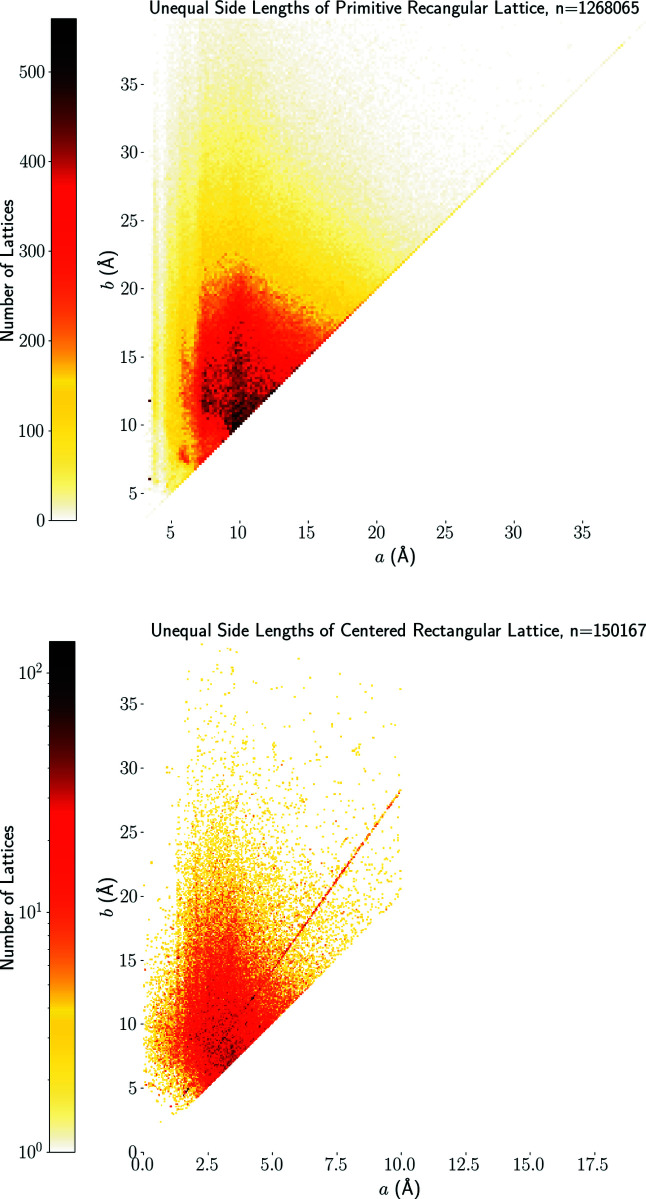
Heat maps of parameters (*a*, *b*) in ångströms. Top: rectangular lattices with primitive unit cells *a* × *b* in *N* = 1 268 065 crystal structures in the CSD. Bottom: centred rectangular lattices with conventional cells 2*a* × 2*b* in *N* = 150 167 crystal structures in the CSD.

The heat map of rectangular lattices in Fig. 11 ▸ (top) has two high-concentration (black) pixels at *a* ≃ 3.5 Å arising from 386 near-identical primitive monoclinic crystal structures of α-oxalic acid dihydrate. This molecule was used as a benchmark for the calculation of electron densities since its crystallographic properties were thoroughly documented by Stevens & Coppens (1980 ▸). Hundreds of publications have since generated and deposited further refinements of its structural determination.

In the heat map of centred rectangular lattices in Fig. 11 ▸ (bottom), the most prominent feature is the hottest area in the region where the shortest side length is between 2.5 and 5 Å. We also see a visible line 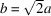 of high-concentration pixels. This line represents two-dimensional lattices in body-centred cubic lattices, where the ratio of side lengths is 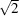 . This ratio was reported among preferred values for lattice length ratios in dimension 3 by de Gelder & Janner (2005 ▸). Another high-concentration pixel represents 130 structures of a standard test molecule (hexamethylenetetramine), which was frequently used in the investigation of lattice vibrations (Becka & Cruickshank, 1963 ▸).

**Figure 12:**
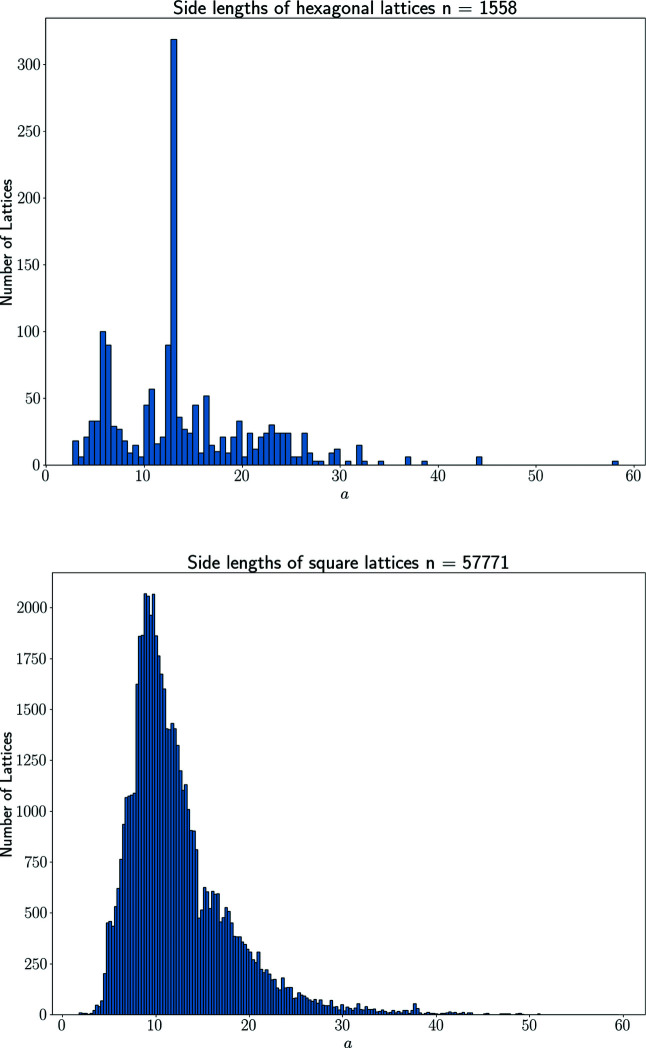
The histograms of minimum inter-point distances *a* in ångströms.

Hexagonal and square lattices are characterized by the inter-point distance *a*. Fig. 12 ▸ shows distributions and preferred values of *a* (in Å) among CSD lattices.

## 5. Other complete invariants and a spherical map of two-dimensional lattices

In comparison with other complete invariants, RI(Λ) has the advantage of homogeneity so that any permutation σ of (indices of) superbase vectors *v*_0_, *v*_1_, *v*_2_ permutes the three root products accordingly: 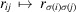 . The metric tensor 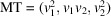 including the coefficients of the form *Q*_Λ_(*x*, *y*) = *q*_11_ *x* ^2^ + 2*q*_12_ *xy* + *q*_22_ *y* ^2^ representing Λ is not homogeneous in the above sense. Taking square roots gives the *quadratic invariant* QI(Λ) = (τ_11_, τ_12_, τ_22_) = 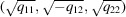 in the units of basis coordinates. The quadratic invariant QI(Λ) is complete up to isometry by Theorem 3.4[Statement theorem3.4](*a*).

In the isosceles triangle QT, continuous metrics and chiral distances have simple formulae in the work of Kurlin (2022*b* ▸, sections 5–6) for the coordinates 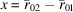 , 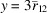 but can be now re-written for any coordinates on 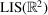 [see the earlier non-isosceles triangles of Engel *et al.* (2004 ▸, Fig. 1.2 on p. 82) and Zhilinskii (2016 ▸, Fig. 6.2)].

Since the quotient square QS = QT^+^ ∪ QT^−^ with identified sides is a punctured sphere, it is natural to visualize QS as the round surface of Earth with QT^±^ as the north/south hemispheres separated by the equator along their common boundary of QT represented by projected invariants PI(Λ) of all mirror-symmetric lattices Λ.

We can choose any internal point of the quotient triangle QT as the north pole. The most natural choice is the incentre *P* ^+^ (pole), the centre of the circle inscribed into QT^+^ because the rays from *P* ^+^ to the vertices of QT^+^ equally bisect the angles 90°, 45°, 45°. The incentre of QT^+^ has the coordinates (*x*, *x*), where 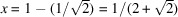 . The lattice 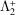 with the projected invariant 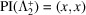 has the basis *v*_1_ ≃ (1.9, 0), *v*_2_ ≃ (−0.18, 3.63) inversely designed by Kurlin [2022*b* ▸, example 4.10 (Λ_2_)].

**Figure 13:**
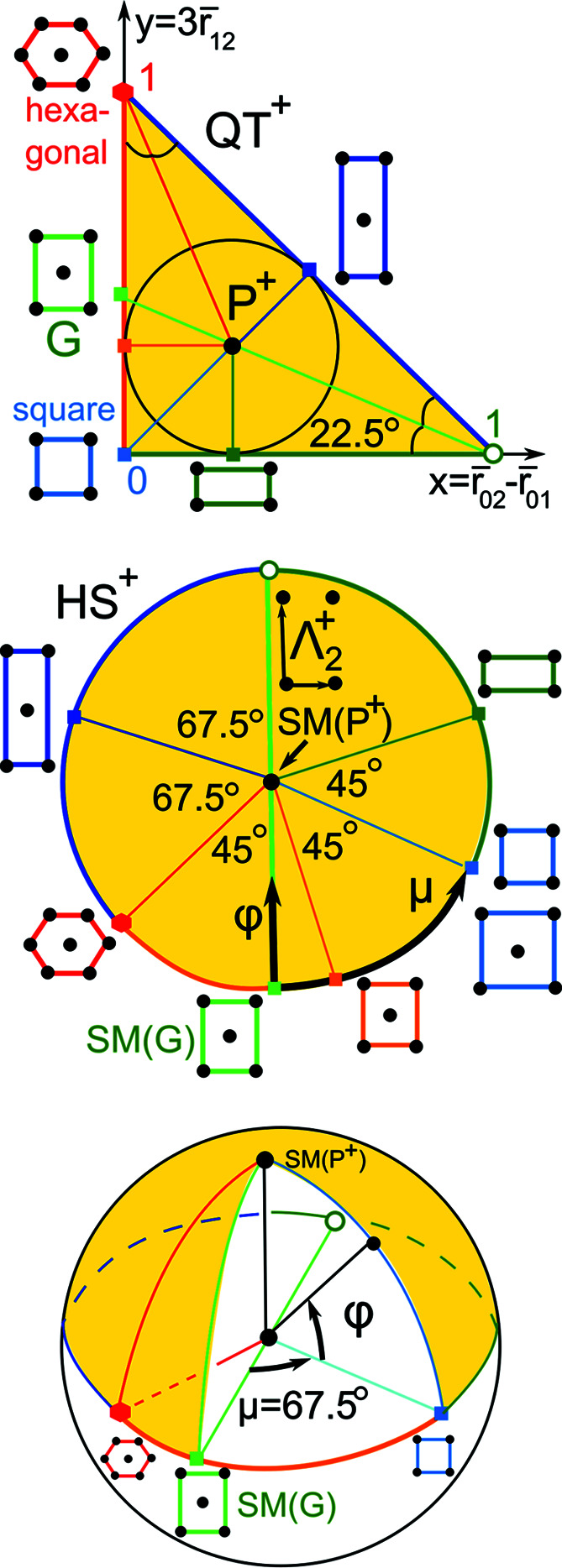
Top: in QT^+^, the Greenwich line goes from the ‘empty’ point (1,0) through incentre *P* ^+^ to the point 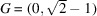 . Middle: the hemisphere HS^+^ has the north pole at *P* ^+^, the equator ∂QT^+^ of mirror-symmetric lattices. Bottom: the longitude μ ∈ (−180°, + 180°] anticlockwise measures angles from the Greenwich line, the latitude φ ∈ [−90°, + 90°] measures angles from the equator to the north pole.

Definition 5.1 (spherical map SM: QS → S2) (*a*) The *spherical map* SM sends the incentre *P* ^+^ of QT to the north pole of the hemisphere HS^+^ and the boundary ∂QT to the equator of HS^+^ [see Fig. 13 ▸ (middle)]. Linearly map the line segment between *P* ^+^ and any point (*x*, *y*) in the boundary ∂QT to the shortest arc connecting the north pole SM(*P* ^+^) to SM(*x*, *y*) in the equator of HS^+^. Extend the *spherical map* to SM: QS → *S* ^2^ by sending any pair of invariants PI^o^(Λ^±^) with sign(Λ^±^) = ±1 to the northern/southern hemispheres of the two-dimensional sphere *S* ^2^, respectively.

(*b*) For any lattice 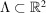 , the *latitude* φ(Λ) ∈ [−90°, + 90°] is the angle from the equatorial plane EP of *S* ^2^ to the radius-vector to the point SM[PI^o^(Λ)] ∈ *S* ^2^ in the upwards direction. Let *v*(Λ) be the orthogonal projection of this radius-vector to EP. Define the *Greenwich* point as 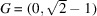 ∈ ∂QT in the line through *P* ^+^ and (1, 0). This *G* represents all centred rectangular lattices with a conventional unit cell 2*a* × 2*b* whose ratio 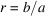 can be found from Example 3.3:

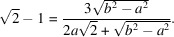

Setting 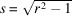 , we get 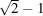 = 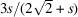 , 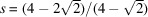 , 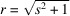 ≃ 1.1. The *Greenwich meridian* is the great circle on the sphere *S* ^2^ passing through the point SM(*G*) in the equator *E*. The longitude μ(Λ) ∈ (−180°, 180°] is the anticlockwise angle from the *Greenwich plane* through the Greenwich meridian to the vector *v*(Λ) above.

For lattices with PI(Λ) in the straight-line segment between the excluded vertex (1, 0) and the incentre *P* ^+^, we choose the longitude μ = +180° rather than −180°. Proposition 5.2[Statement proposition5.2] computes μ(Λ), φ(Λ) via PI(Λ) = (*x*, *y*) and is proved in Appendix *A* [App appa].

Proposition 5.2 (formulae for SM) For any lattice 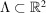 with PI(Λ) = (*x*, *y*) ∈ QT, if 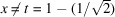 , then set 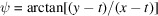 , otherwise ψ = sign(*y* − *t*)90°.

The longitude of the lattice Λ is

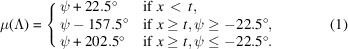

The latitude is

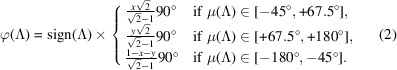

The incentres *P* ^±^ ∈ QT^±^ have ψ = 0 and φ = ±90°, respectively, μ is undefined.

*Example 5.3 (prominent lattices)*. Any mirror-symmetric lattice 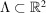 has sign(Λ) = 0, and hence belongs to the equator *E* of *S* ^2^ and has φ(Λ) = 0 by (2). Any square lattice Λ_4_ with PI(Λ_4_) = (0, 0) has 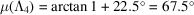 by (1). Any hexagonal lattice Λ_6_ with PI(Λ_4_) = (0, 1) has 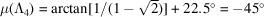 . Any rectangular lattice Λ with 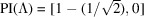 has μ(Λ) = −90° + 202.5° = 112.5°. Any centred rectangular lattice Λ with 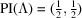 at the midpoint of the diagonal of QT has 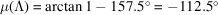 . Any *Greenwich* lattice Λ_*G*_ with PI(Λ_*G*_) = *G* = 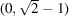 has 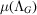 = 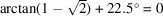 .

**Figure 14:**
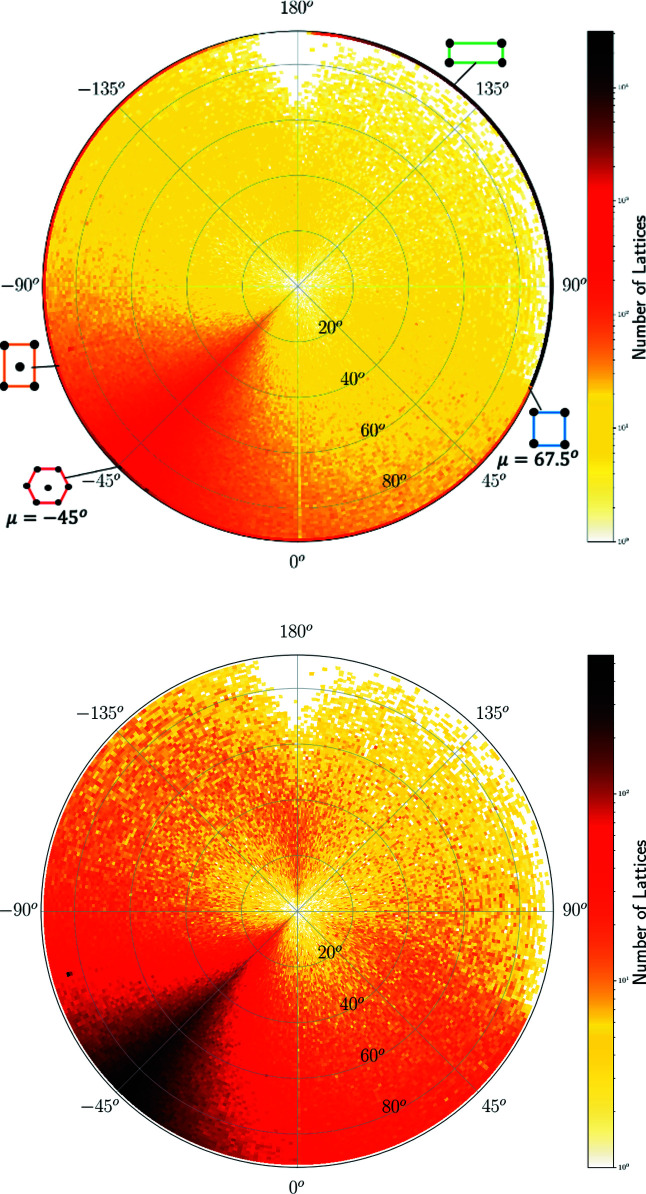
The heat map of two-dimensional lattices from crystal structures in the CSD on the northern hemisphere. The radial distance is the latitude φ ∈ [0°, 90°]. Top: all *N* = 2 191 887 lattices with sign(Λ) ≥ 0, φ ≥ 0. Bottom: all *N* = 741 105 oblique lattices with sign(Λ) > 0, φ > 0.

The north pole represents the incentre *P* ^+^ whose pixel contains 230 lattices in Fig. 10 ▸ but appears sparsely populated in Fig. 14 ▸ because this incentre pixel is split into many 1 × 1° curved ‘pixels’ of a much lower concentration. The high concentration near the point representing hexagonal lattices is visible in Figs. 14 ▸, 15 ▸ as dark pixels near the longitude μ = −45°. Where non-oblique lattices are included, we see the high concentrations along the borders of QT, with primitive rectangular lattices appearing as a dark thick arc on the equator for μ ∈ [67.5°, 180°).

**Figure 16:**
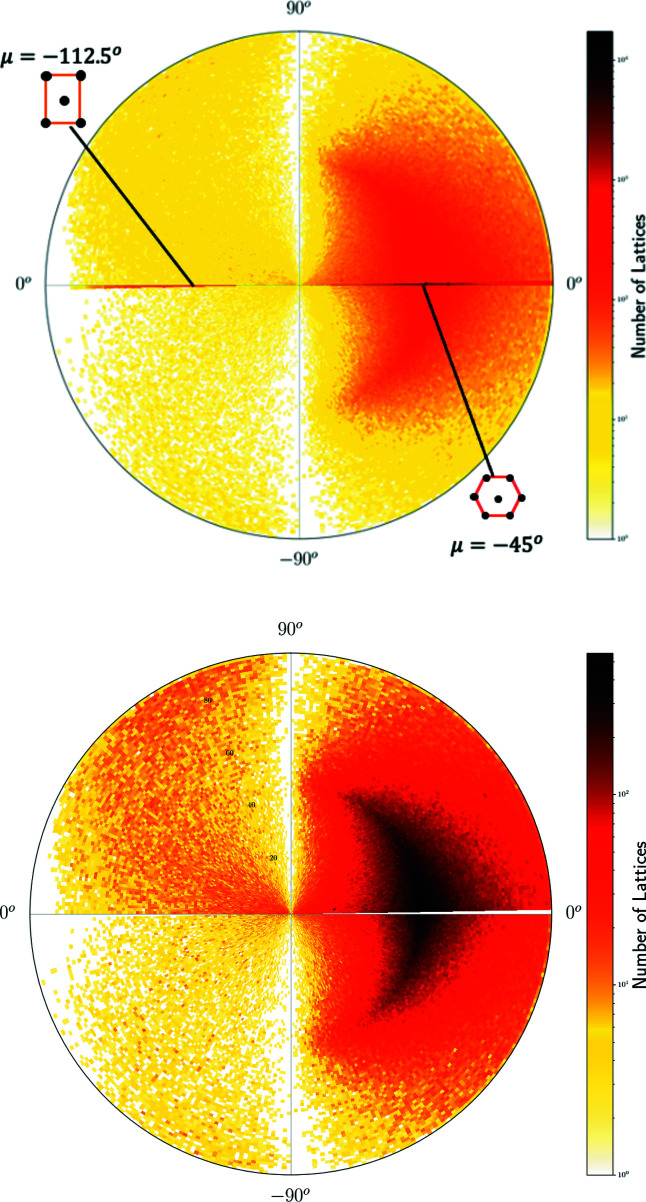
The heat map of two-dimensional lattices from crystal structures in the CSD on the western hemisphere. Angles on the circumference show the latitude φ ∈ [−90°, 90°]. Top: *N* = 1 100 580 lattices with μ ∈ (−180°, 0°]. The hexagonal lattice at μ = −45° and the centred rectangular lattice at μ = −112.5° are marked on the horizontal arc (western half-equator). Bottom: all *N* = 932 626 oblique lattices with μ ∈ (−180°, 0°] and φ ≠ 0.

The heat maps show a hexagonal ‘ridge’ along the meridional arc at μ = −45° in Figs. 14 ▸ and 15 ▸, which appears as a round arc in Figs. 16 ▸ and 17 ▸. The concentration of exact square and rectangular lattices is even higher (dark pixels for the Bravais classes tp and op), but there are fewer lattices close to these classes possibly because manual or automatic adjustments are easier for angles close to 90° than to 60°.

## 6. Main conclusions and motivations for a continuous crystallography

The heat maps in Figs. 9 ▸–10 ▸ and 14 ▸–17 ▸ visualize for the first time 2.6 million two-dimensional lattices in real crystal structures from the CSD. The preprint of Bright *et al.* (2021 ▸) extends this approach to three-dimensional lattices, but there is a growing database of real and theoretical two-dimensional lattice structures with potentially interesting properties (Mounet & Gibertini, 2020 ▸) for which two-dimensional lattice invariants may have direct utility. The maps indicate that lattices occur naturally in continuous distributions, and their geometry can be investigated by continuous invariant-based classification in addition to using discrete symmetry groups. The continuous approach has the added advantage of more easily spotting structures that are geometrically nearly identical, but where small variances in crystallization conditions have led to slight structure perturbations which disrupt higher lattice symmetries. The Python code for new invariants is available at https://github.com/MattB-242/Lattice_Invariance.

Using a geographic analogue, the recent isometry invariants create complete and continuous maps for efficient navigation in the lattice isometry space 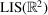 , which can be magnified as satellite images and explored at any desirable resolution. Since each invariant is a point in a space on which various metrics can be defined, this representation leads to a continuous ‘distance’ between two lattices based on their separation in 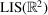 and also a continuous measure of ‘dissymmetry’ as the closest distance to the subspace corresponding to lattices with higher symmetry (see Kurlin, 2022*b* ▸).

The four non-generic Bravais classes of two-dimensional lattices are lower-dimensional subspaces in 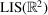 whose separate maps in Fig. 11 ▸ and 12 ▸ have no intermediate gaps and include sparse or empty regions only for small or very large values of cell parameters.

Using a biological analogue, crystallography previously took a similar approach to the classical taxonomy, dividing lattices into an increasingly complex sequence of discrete categories based on symmetries as they divided organisms according to physical characteristics; see a comprehensive review by Nespolo *et al.* (2018 ▸).

The new area of *continuous crystallography* uses the geometric properties of the lattice itself to continuously classify an individual lattice in as granular a manner as we like, in a manner akin to the modern use of genetic sequences and markers to classify organisms. Indeed, since the root invariant RI(Λ) of a lattice Λ is complete, this RI(Λ) could be said to represent the DNA of Λ. Even better than the real DNA, any two-dimensional lattice can be explicitly built up from RI(Λ) [see Kurlin (2022*b* ▸), proposition 4.9].

The complete root invariant from Definition 3.1[Statement definition3.1] extends to a three-dimensional lattice as follows. For any three-dimensional lattice, depending on its Voronoi domain, all obtuse superbases 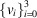 with *v*_0_ + *v*_1_ + *v*_2_ + *v*_3_ = 0 are described by Kurlin (2022*a* ▸, lemmas 4.1–4.5). Any generic three-dimensional lattice has a unique (up to isometry) obtuse superbase whose root products *r*_*ij*_ = 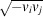 can be considered as labels on the edges of a three-dimensional tetrahedron or written in the matrix

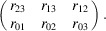

Permutations of four superbase vectors induce 4! = 24 permutations of the above six root products. Other non-generic cases require other permutations, which were not previously considered by Andrews *et al.* (2019*b* ▸), to guarantee a complete invariant of all three-dimensional lattices [in Kurlin (2022*a* ▸, theorem 6.3)]. Maps of three-dimensional lattices extracted from crystal structures in the CSD appear in the work of Bright *et al.* (2021 ▸).

Working towards a complete materials genome, Widdowson *et al.* (2022 ▸, section 7) introduced the pointwise distance distribution (PDD). This PDD invariant distinguished all periodic point sets after a tiny perturbation. More than 200 billion pairwise comparisons of all 660 000+ periodic crystal structures in the CSD over 2 days on a modest desktop PC detected five pairs of isometric duplicates [see Widdowson *et al.* (2022 ▸), section 7], where two crystals are geometrically identical to the last decimal place in all data including structure factors but one atom is replaced with a different one: Cd with Mn in the pair HIFCAB versus JEPLIA. These pairs are under investigation by five journals for data integrity. (Near-)duplicates in the CSD can be recognized only by a *continuous* invariant taking close values for close crystals. The CSD entries DEBXIT01,…, DEBXIT06 represent two polymorphs: four (near-)duplicates of T2-γ and two (near-)duplicates of T2-β reported in our past work (Pulido *et al.*, 2017 ▸). Zhu *et al.* (2022 ▸) predicted and synthesized new material based on PDD invariants.

## Figures and Tables

**Figure 15:**
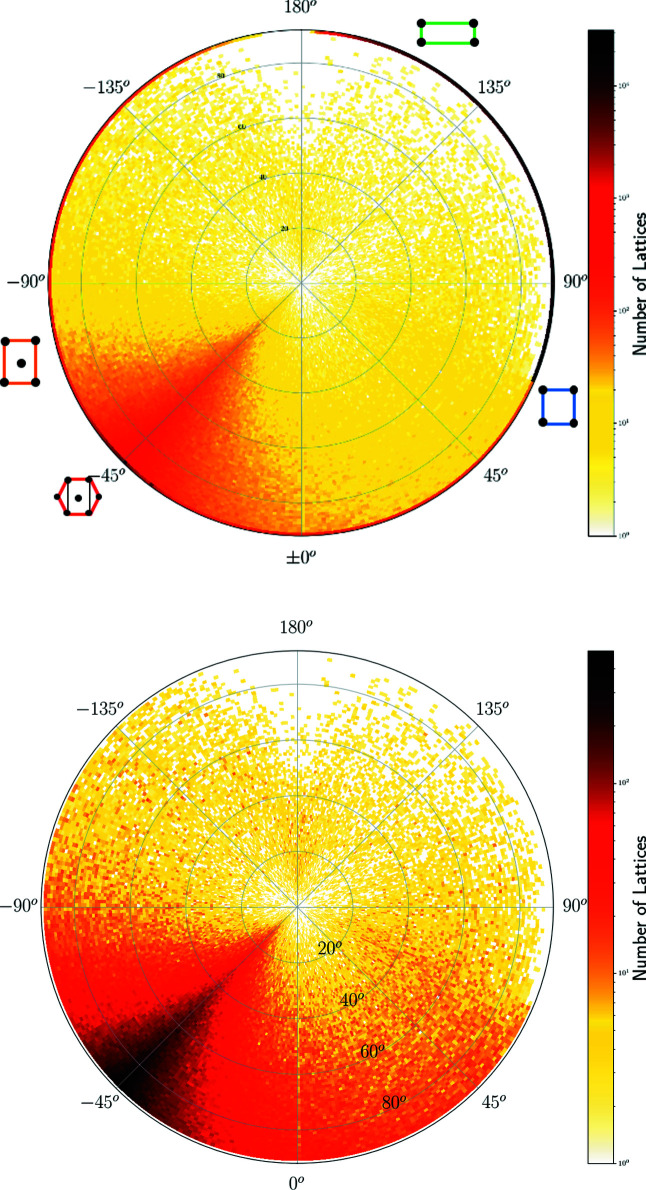
The heat map of two-dimensional lattices from crystal structures in the CSD on the northern hemisphere. The radial distance is the latitude φ ∈ [0°, 90°]. Top: all *N* = 1 854 209 lattices with sign(Λ) ≤ 0, φ ≤ 0. Bottom: all *N* = 406 930 oblique lattices with sign(Λ) < 0, φ < 0.

**Figure 17:**
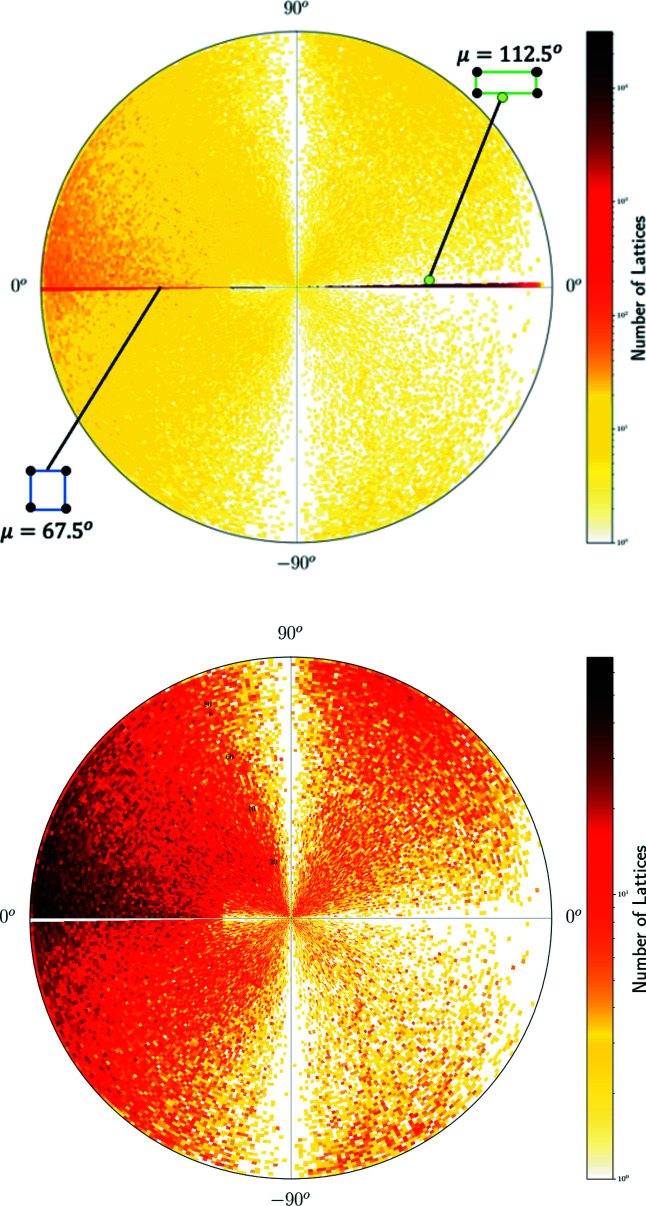
The heat map of two-dimensional lattices from crystal structures in the CSD on the eastern hemisphere. Angles on the circumference show the latitude φ ∈ [−90°, 90°]. Top: all *N* = 1 511 307 lattices with μ ∈ [0°, 180°), the square lattice point at μ = 67.5° and the rectangular lattice at μ = 112.5° are marked on the horizontal arc (eastern half-equator). Bottom: all *N* = 215 409 oblique lattices with μ ∈ [0°, 180°), φ ≠ 0.

**Figure 18:**
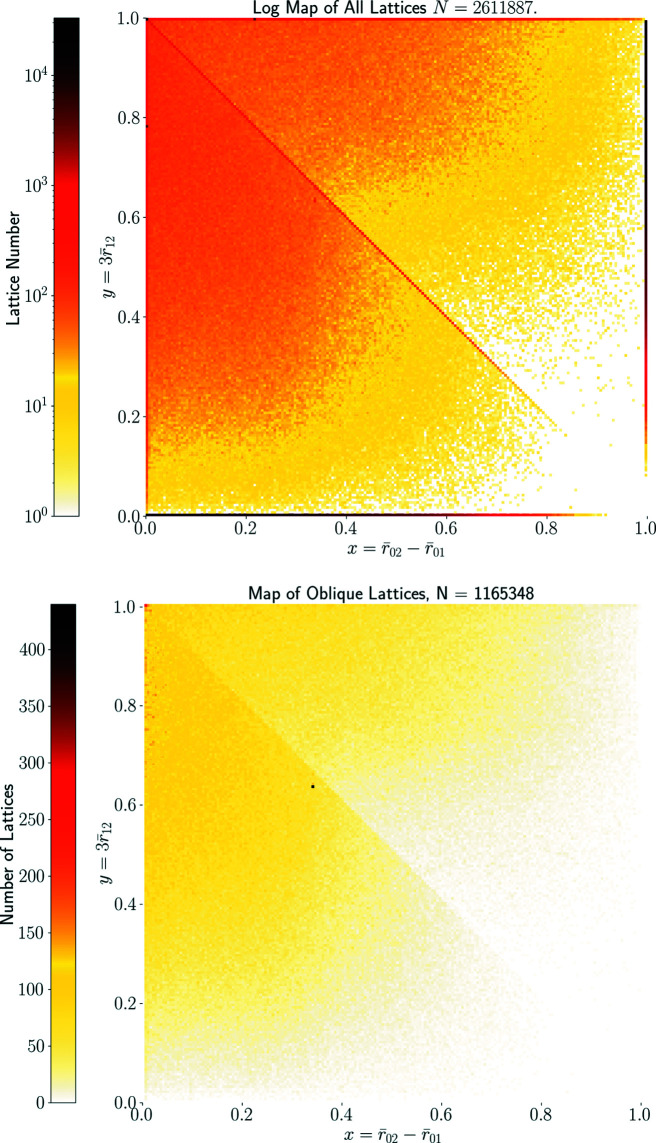
Heat maps of two-dimensional lattices derived from crystal structures in the CSD in the quotient square QS. Each pixel in the map represents a 0.005 × 0.005 interval of projected form invariant value, where each such value uniquely represents a lattice up to rigid motion only. Top: *N* = 2 611 887 lattices derived from the CSD. Projected invariants for primitive and centred rectangular lattices are duplicated at the boundaries of the quotient square – indicative positions of non-trivially symmetric lattices are shown. Bottom: all *N* = 1 165 348 oblique (non-mirror-symmetric) lattices derived from the CSD.

## Appendix A A proof of Proposition 5.2[Statement proposition5.2] and plots of orientation-aware invariants

(*a*) For any point *P* = (*x*, *y*) ∈ QT, the vector  has coordinates (*x* − *t*, *y* − *t*), where *P* ^+^ = (*t*, *t*) is the incentre (the centre of the inscribed circle) of the quotient triangle QT and  [see Fig. 13 ▸ (top)]. Recall that, for any  , the function  outputs a unique angle α ∈ (−90°, 90°) such that  . If *x* > *t*, then  =  is the anticlockwise angle from the positive *x* direction (with the origin at *P* ^+^) to the vector  .

For *x* = *t*, the limit values of arctan give ψ = sign(*y* − *t*)90°. For *x* < *t*, the anticlockwise angle from the positive *x* direction to  is ψ + 180°. For example, the Greenwich vector  from the excluded vertex (1, 0) to  ∈ QT has the anticlockwise angle ψ + 180° = 157.5° from the positive *x* direction because

and  . The anticlockwise angle from the *x* axis to  is

In all cases above, since the Greenwich vector  was chosen as the 0-th meridian, the anticlockwise angle from  to  is the longitude μ = α − 157.5°. For example, any centred rectangular lattice Λ with  has  =  =  and longitude μ = α − 157.5° = −112.5°. If α − 157.5° is outside the expected range of μ ∈ (−180°, 180°], we add or subtract 360°. Any hexagonal lattice Λ_6_ with PI(Λ_6_) = (0, 1) has

α = ψ + 180° = 112.5° and longitude μ = α − 157.5° = −45°. Any square lattice Λ_4_ with PI(Λ_6_) = (0, 0) has  =  , α = ψ + 180° = 225° and longitude μ = α − 157.5° = 67.5°. Equation (1) is split into three subcases only to guarantee the range of a longitude μ ∈ (−180°, 180°] for the anticlockwise angle α − 157.5° from  to  , where α is computed above.

(*b*) For a fixed longitude μ(Λ), the projected invariant PI(Λ) varies along the line segment *L* at a fixed angle from the incentre *P* ^+^ to the boundary ∂QT. Equation (2) is split into three subcases according to the three boundary edges of QT.

Consider the vertical edge between hexagonal and square lattices, where μ(Λ) ∈ [−45°, 67.5°]. The latitude φ(Λ) is proportional to the ratio in which the point PI(Λ) = (*x*, *y*) splits the line segment *L* from *P* ^+^ to the vertical edge. The endpoint *x* = 0 means that SM[PI(Λ)] is in the equator with φ = 0. The endpoint  means that PI(Λ) = *P* ^+^ is in the centre whose image SM(*P* ^+^) is the north pole with φ = 90°. The linear map between these extreme cases gives  . The case of the horizontal edge of QT gives a similar φ after replacing *x* with *y*. The hypotenuse of QT, where *x* + *y* = 1, is also similar as the incentre

has the latitude

as expected. The factor sign(Λ) in (2) guarantees a symmetry of SM: QS → *S* ^2^ in the equator.

□

In the main body of this paper, we show heat maps of orientation-unaware projected invariants, which clearly demonstrate the way that lattices generated from the CSD distribute through the lattice invariant space without gaps. Fig. 18 ▸ shows plots of orientation-aware projected invariants PI^o^(Λ) for the same data set.

In both plots, we see an additional apparent non-smooth jump across the diagonal representing higher-symmetry lattices, so that there is some apparent favouring of positive chirality among two-dimensional lattices. This is an artefact of the interaction between vectors in the initial CSD data, and our consistently ordered selection of pairs from those vectors, and should not be read as a real physical effect. We also note that there is a much lower relative concentration, apparent from the lightness of the colour of each pixel, in the standard plot of oblique lattices. In this case the oxalic acid structures mentioned in the main body of the paper all have consistent chirality and remain below the diagonal of the quotient square, while the lattices in any other pixel split between each half of the plot and therefore have much lower relative counts.
